# Micro-CT Evaluation of Different Root Canal Irrigation Protocols on the Removal of Accumulated Hard Tissue Debris: A Systematic Review and Meta-Analysis

**DOI:** 10.3390/jcm11206053

**Published:** 2022-10-13

**Authors:** Ailin Liang, Luo Huang, Baoyu Li, Yihua Huang, Xiaoyan Zhou, Xufang Zhang, Qimei Gong

**Affiliations:** 1 Hospital of Stomatology, Sun Yat-sen University, No. 56, Lingyuan West Road, Guangzhou 510055, China; 2 Guangdong Provincial Key Laboratory of Stomatology, No. 74 2nd Zhongshan Road, Guangzhou 510080, China; 3 Guanghua School of Stomatology, Sun Yat-sen University, No. 74 2nd Zhongshan Road, Guangzhou 510080, China; 4 Faculty of Medicine and Health, School of Dentistry, University of Sydney, Sydney, NSW 2006, Australia

**Keywords:** endodontics, accumulated hard tissue debris (AHTD), meta-analysis, micro-CT, root canal irrigation

## Abstract

Accumulated hard tissue debris (AHTD) is an inevitable by-product during endodontic treatment and is difficult to remove completely using traditional syringe and needle irrigation (SNI). Adjunctive irrigation is proposed to assist the clean-up of AHTD. This systematic review and meta-analysis aimed to evaluate the AHTD removal efficacy of different root canal irrigation devices using micro-computed tomography (Micro-CT). A literature search was carried out within the main scientific databases until 20 June 2022. All results were screened with detailed eligibility criteria. Eleven studies were included for analysis. SNI, passive ultrasonic irrigation (PUI), negative pressure systems, sonically activated irrigation (SAI), mechanical-activated system and laser-activated irrigation (LAI) were assessed. PUI is superior to SNI for debris removal and LAI has better AHTD removal performance than PUI. The negative pressure system and mechanical-activated system were proved to be less effective. Registration: PROSPERO (CRD42021273892).

## 1. Introduction

Endodontic therapy is currently considered as the conventional technique for treating pulpal disease. Its success depends on broader principles, for which sufficient mechanical preparation, thorough disinfection and obturation with no voids are important [1,2]. Accumulated hard tissue debris (AHTD) is produced during the mechanical preparation process and pile up in the root canal system, which usually compromise the success of endodontic treatment [3,4]. Research has shown that root canal irrigation helps to remove AHTD; however, no irrigation protocol has been found to completely remove AHTD due to the anatomical complexity of the root canal system, especially in isthmus and apical regions [5,6].

Traditional root canal treatment adopts a manual irrigation approach (syringe and needle irrigation, SNI). SNI is a positive pressure system and the needle tip usually reaches 1 to 2 mm away from the working length (WL). By increasing the irrigation pressure inside the root canal, the effect of debris removal is enhanced [7], while the risk of apical extrusion of the irrigant increases as well [8], which would cause severe complications, such as ‘hypochlorite accident’. At present, needles with a lateral opening, such as NaviTip needle and NaviTip FX (Ultradent, South Jordan, UT, USA), are used to minimize extrusion.

Passive ultrasonic irrigation (PUI) was firstly put forward by Weller et al. [9] and is now widely used in the clinic. “Passive” refers to the “non-cutting” effect, where the ultrasonic file moves freely in the canal without making contact with the canal wall. Acoustic energy is transmitted to the irrigation solution and induces the generation of acoustic streaming to help canal cleaning [10]. Further, ultrasound can break the bubble resistance in the apical region and promote a more efficient distribution of the irrigant inside the complex root canal system [11].

A negative pressure irrigation system is proposed to solve the issue of irrigant extrusion out of apical foramen. The EndoVac (EV, Discus Dental, CA) system is a typical negative pressure system consisting of two cannulas and one master delivery tip. Irrigant is delivered into the chamber via the master delivery tip. Macro-Cannula and Micro-Cannula are used sequentially to aspirate the solution from the microscopic evacuation holes through negative pressure [12].

The sonically activated irrigation (SAI) system sonically powers a flexible polymer tip to vibrate at high amplitude in the root canal in an oscillating motion. Representative equipment includes EndoActivator (EA, Dentsply Sirona, Charlotte, NC, USA), EDDY (ED, VDW, Munich, Germany) and Vibringe (Vibringe BV, Amsterdam, The Netherlands). EA consists of two parts: a wireless handpiece and a working tip. ED is designed to generate mechanical vibrations at the polyamide tip by providing high-frequency power up to 6000 Hz. Vibringe is a combination of manual delivery and sonic cavitation. Irrigation solution is delivered by a wireless handpiece connected to a conventional syringe and cavitation is added under the control of a microprocessor system.

The mechanical-activated system is another widely used irrigation device. XP-Endo Finisher (XPF, FKG Dentaire, Le Crêt-du-Locle, Switzerland) is characteristic with a flexible rotary Ni-Ti file. The file is linear at room temperature and broadens into a sickle shape at body temperature. This feature allows the file to better adapt to root canal anatomy and minimize the destruction on the root structure [13]. Easy Clean (Easy Dental Equipment, Belo Horizonte, Brazil) is similar to the root canal preparation instrument and has a wing-shaped working file. Self-adjusting file (SAF) is a hollow Ni-Ti instrument that can be put in the prepared root canal without a hitch and tend to recover its original shape, allowing for continuous gentle pressure on the root canal wall.

Laser-activated irrigation (LAI) relies on cavitation to clean up root canals. Radiation is transmitted to a solution and promotes the generation, expansion and burst of bubbles. In addition, secondary cavitation and acoustic streaming are induced by subsequent laser pulses [14]. Photon-induced photoacoustic streaming (PIPS) uses a low energy level (10~20 mJ) and short pulse duration (50 μs). Unlike traditional methods, it does not require the tip to be placed into the root canal, instead resting it at the root canal orifice or pulp chamber. It reduces the need of extended preparation for access and avoids damage on the dentin due to the laser-generated thermal energy. Shock-wave-enhanced emission photoacoustic streaming (SWEEPS) is the latest development of LAI using a double pulse to create a more intense shock wave. Conflicting results remain in current research on the cleaning effect of SWEEPS. Yang et al. [15] evaluated the AHTD removal effects of PUI, PIPS and SWEEPS using Micro CT and found that SWEEPS had a greater reduction in AHTD compared with the PIPS and PUI groups. Kirmizi et al. [16] reported that SWEEPS and PIPS were significantly better than PUI in calcium hydroxide removal, while there was no significant difference between SWEEPS and PIPS groups. However, Mancini et al. [17] used field emission scanning electron microscopy (FESEM) to evaluate endodontic smear layer removal from root canals and found that PIPS was more efficient than SWEEPS in terms of smear layer reduction.

Currently, different methods have been used to evaluate the cleaning efficacy of irrigation, including weight determination, histological assessment, scanning electron microscope (SEM) and micro-computed tomography (micro-CT). Weight measurement was firstly proposed by McKendry et al. [18] and is mainly used to evaluate the degree of apical debris extrusion. Briefly, debris extruded from the apical foramen are collected onto a pre-weighed container and then weighted on an analytical electrobalance after desiccation. The histological method has been used in endodontic research for decades since Vansan et al. [19] firstly used it to quantify the residual debris remaining in the root canals after irrigation. The tooth samples are fixed with 10% neutral buffered formalin, then decalcified and embedded in paraffin. Serial cross-sections are cut and stained with hematoxylin and eosin or tricolor Gomori alternately and, finally, observed under an optical microscope [20,21]. SEM is mainly used for the evaluation of smear layer and debris. Prepared teeth are longitudinally bisected, desiccated and then coated with gold to obtain microphotographs [22]. The smear layer and debris are evaluated following the criteria developed by Caron et al. [23] and Dadresanfar et al. [24]. However, a previous study stated that the score-based results obtained from SEM are unreliable and non-reproducible because the debris and filling material displace during the specimen handling process [25]. Since teeth were destroyed during the handling process, it is impossible to compare the pre- and post-irrigation status on the same tooth, so only a pair of similar teeth can be used to represent the status before and after irrigation [26]. Moreover, some tooth samples fractured or cracked during the handling process due to technical problems, resulting in the loss of experimental data [27].

Micro-CT is a non-destructive method to assess the three-dimensional shape of root canals and the distribution of debris or filling material [28,29]. Teeth can be screened multiple times during treatment and there is no risk of data loss. This feature allows the researchers to measure changes in root canal volume after different sequential treatment phases on the same tooth [30]. Micro-CT was employed to compare the root canal morphology before and after preparation to investigate the shaping and debridement ability of different instruments and techniques. Substances with a density similar to dentin in regions previously occupied by air in the pre-instrumented root canal were identified as debris and quantified by intersection between images before and after irrigation [31,32].

Although there have been investigations on the debridement ability of various activated irrigation techniques, conflicting results were reported from different studies. A gap still exists in understanding which approach is better for AHTD removal. Thus, we aimed to conduct a meta-analysis to evaluate the effect of different irrigation protocols in removing AHTD, which would help to develop an evidence-based protocol for root canal irrigation.

## 2. Methods

This meta-analysis was undertaken according to the PRISMA guidelines [33] and the Cochrane Handbook (handbook.cochrane.org). The project was registered with the International Prospective Register of Systematic Review (PROSPERO CRD42021273892). This study aimed to assess the impact of different irrigation protocols on the removal of AHTD. The detailed PICOS principles were as follows: 1. Participants: extracted human teeth with complete root formation and no obvious root caries, root crack or root absorption. 2. Intervention: canal irrigation with different adjunctive irrigation protocols. 3. Control: canal irrigation with PUI. 4. Outcome: percentage reduction in AHTD after irrigation. 5. Study design: observational study.

### 2.1. Search Strategy

PubMed, Web of Science, Embase, Cochrane and Scopus were searched for articles published up to 20 June 2022 without any restriction in terms of countries or article types. An adequate search strategy was used in each database with search string shown in Supplementary Table S1. Manual searches of citation list of all retrieved articles were conducted. Opengrey, OpenDoar, OpenAire and Base were searched for grey literature.

### 2.2. Eligibility Criteria

Studies were selected based on the following inclusion criteria:

1. The subjects were extracted human teeth with complete root formation and no obvious root caries, root crack or root absorption.
2. The root canal was cleaned with the irrigation protocols as described in the Introduction.
3. AHTD in the root canal was evaluated by micro-CT before and after adjunctive root canal irrigation.
4. The percentage reduction in AHTD after irrigation could be obtained directly or indirectly from the outcomes of interest.
5. The study was an observational study.
6. The study was published in English.

Studies were excluded based on the following criteria:

1. Full-text article was not available.
2. Artificial grooves or animal models were used as subjects.
3. Lacking comparison with PUI in the study.
4. Case reports, review articles and critical appraisal articles.

### 2.3. Study Selection and Data Collection

EndNote 20 software (Clarivate, London, UK) was used to sort out records retrieved from different databases and remove duplicates. Titles and abstracts of all studies were screened independently by two authors (Liang and Huang). For potentially eligible studies, full texts were reviewed for eligibility based on the inclusion criteria by two authors independently. Disagreements were resolved by discussion with senior authors (Huang, Zhou, Zhang and Gong). Data from all included studies were extracted separately by two authors and agreement was reached through discussion with other reviewers (Huang, Zhou, Zhang and Gong) when there were disagreements. The primary outcome was the mean and standard deviation (SD) of AHTD reduction percentage after adjunctive irrigation.

### 2.4. Quality Assessment

Two reviewers independently assessed the quality of selected studies according to the Joanna Briggs Institute (JBI) critical appraisal tool for observational studies. Two evaluators (Liang and Huang) independently assessed eligible studies and scored each JBI question as yes, no or unclear, to obtain the risk of bias. Discrepancies in assessment were discussed with senior endodontists (Huang, Zhou, Zhang and Gong) until a consensus was reached. The final score of each article subjected to the JBI appraisal was calculated based on the percentage of positive answers (‘yes’) and was classified as having a ‘high’ risk of bias when the score was ≤49%, ‘moderate’ risk of bias if the score was 50–69% and ‘low’ risk of bias if the score was >70%.

### 2.5. Statistical Analysis

For studies only reporting the volume of debris obtained before (A) and after (B) the final irrigation, the percentage debris reduction (red%) was calculated according to the following formula: red% = 100% ∗ (A − B)/A, and the SD of baseline was used as an estimate for the SD of the reduction percentage, as described in Kirwan et al. [34]. In the absence of other information, we assumed a conservative 0.5 correlation between the follow-up and baseline measurements. Between-study heterogeneity was assessed by Q and I^2^ test. Heterogeneity is considered to exist when I^2^ > 50% or *p* < 0.1 and random-effects model was used for data analysis; otherwise, fixed-effects model was used. In addition, Begg’s test was used to determine the presence of publication bias. A two-tailed *p* value of less than 0.05 was considered statistically significant.

### 2.6. Grading of the Evidence

GRADEpro was used for the grading of evidence. Quality degradation factors include risk of bias, inconsistency, indirectness, imprecision and publication bias (minus 1 point for severe and 2 points for extremely severe). Escalation factors include large effect (plus 1 point, plus 2 points), plausible confounding factors that reduce efficacy (plus 1 point), dose–effect relationship: drug dose and effect size are significantly correlated (plus 1 point). Finally, quality evidence was graded as “high ++++”, “moderate +++”, “low ++” and “very low +”.

## 3. Results

**Figure 1:**
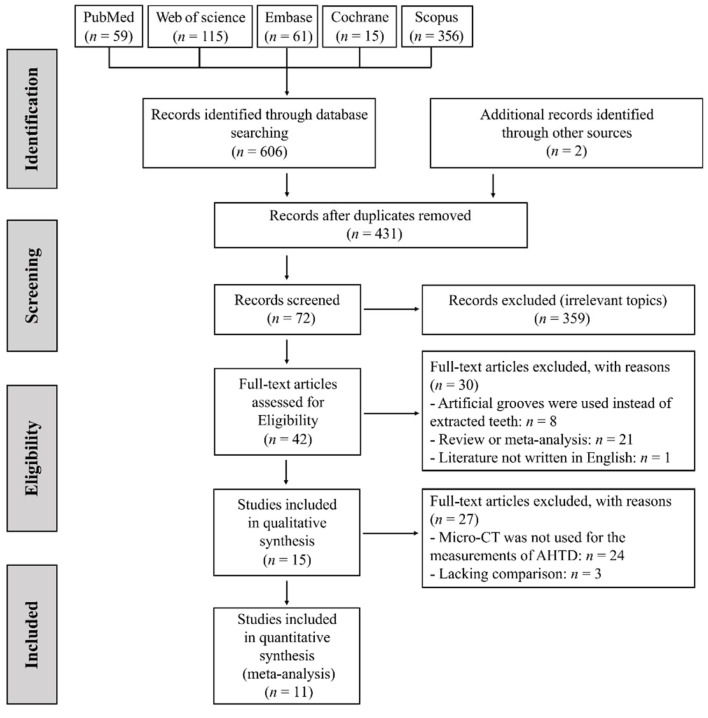
PRISMA flow chart of study selection.

A total of 606 studies and 2 grey studies were retrieved and 431 studies remained after the removal of duplicates. After screening of titles and abstracts, 359 articles were excluded for irrelevant topic, 8 for using artificial grooves instead of extracted human teeth, 21 for reviews or meta-analysis and 1 for not being written in English. The remaining 42 articles were screened and evaluated by two reviewers independently. In total, 27 studies were further excluded for the following reasons: 3 for lacking comparison and 24 for using SEM or histological methods for evaluation rather than Micro-CT. Finally, eleven studies were included for meta-analysis [15,29,35,36,37,38,39,40,41,42,43]. The literature screening process is shown in Figure 1. References listed in the eligible articles were examined and no additional eligible studies were identified.

### 3.1. Characteristics of the Included Studies

The main characteristics of the included studies are listed in Table 1. All studies used extracted human teeth without pathological change as subjects, and PUI were used as the control group. Mandibular molars were adopted in ten studies and mandibular incisors were used in one. The curvature of the root canal ranged between 0 and 46°. Irrigants employed in all studies were NaOCl, EDTA and saline solution, which have been widely used in the clinic. Micro-CT was used in all research before and after adjunctive root canal irrigation and the percentage reduction in AHTD after irrigation can be obtained as the outcome of interest.

### 3.2. Risk-of-Bias Judgement of Eligible Studies

**Figure 2:**
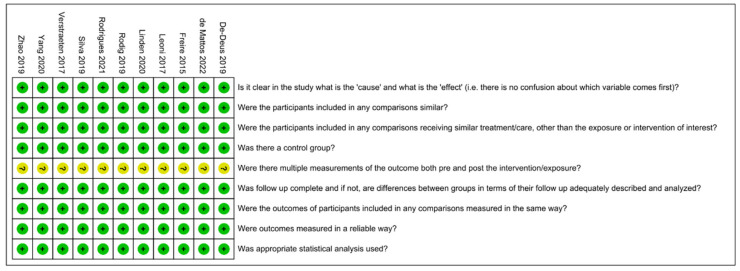
Risk-of-bias summary. Green: low risk of bias; Yellow: unclear risk of bias [15,29,35,36,37,38,39,40,41,42,43].

**Figure 3:**
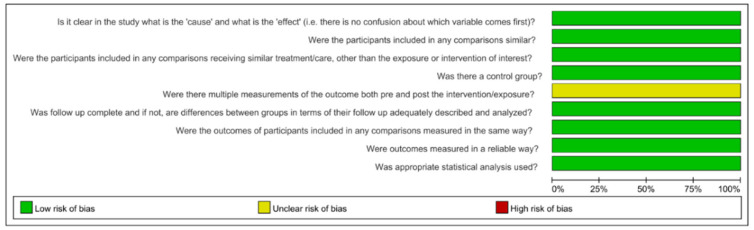
Risk-of-bias graph.

The assessment of risk of bias is shown in Figure 2 and Figure 3 [15,29,35,36,37,38,39,40,41,42,43]. All 11 included studies were classified as having a low risk of bias, with an average score of 88.90%. All studies made the intervention and outcomes clear. No samples in any included studies received similar treatment other than the intervention of interest. All studies have a group treated by PUI as a control group. No study mentioned if multiple measurements of the outcome were conducted before and after the irrigation.

### 3.3. Outcomes of the Meta-Analysis and Publication Bias

#### 3.3.1. Passive Ultrasonic Irrigation System

**Figure 4:**
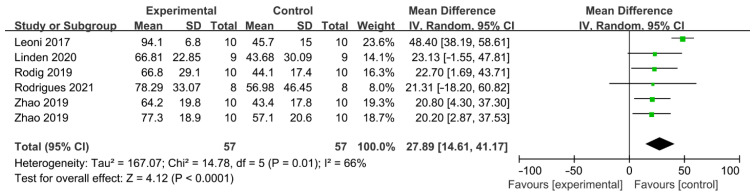
Forest plot and meta-analysis of comparison between PUI and SNI [35,36,37,39,42].

The difference in cleaning efficacy between PUI and SNI was reported by five articles as shown in Figure 4 [35,36,37,39,42] and a total of 114 teeth was included. Each study included one comparison, except Zhao, which included two independent comparisons. PUI showed better AHTD removal effect than SNI (WMD: 27.89, 95% CI: 14.61 to 41.17; *p* < 0.0001) with significant between-study heterogeneity (χ^2^ = 14.78, df 5, *p* = 0.01, I^2^ = 66%). No publication bias was detected by Begg’s test (*p* = 0.75).

#### 3.3.2. Negative Pressure Irrigation System

**Figure 5:**
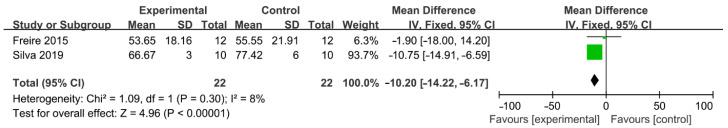
Forest plot and meta-analysis of comparison between negative pressure irrigation system and PUI [29,38].

Comparation of EndoVac and PUI was available in two studies and 44 teeth were involved totally (Figure 5) [29,38]. EV was found to be less effective than PUI regarding to AHTD removal (WMD: −10.20, 95% CI: −14.22 to −6.17; *p* < 0.00001) and no significant heterogeneity between studies was found (χ^2^ = 1.09, df 1, *p* = 0.30, I^2^ = 8%). There was evidence of publication bias detected by Begg’s test (*p* < 0.01).

#### 3.3.3. Sonically Activated Irrigation System

**Figure 6:**
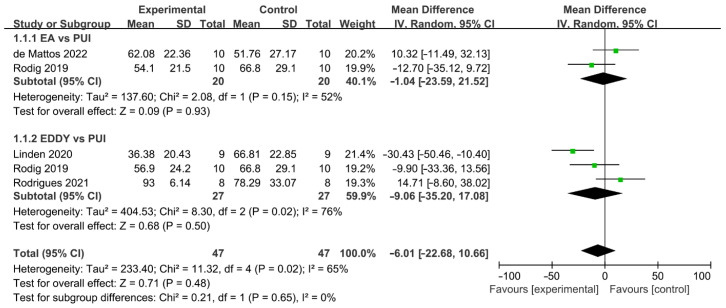
Forest plot and meta-analysis of comparison between SAI and PUI [35,36,39,43].

The difference in cleaning efficacy between SAI and PUI was reported by four studies (Figure 6) [35,36,39,43]. The overall effect showed no significant difference between the two groups (WMD: −6.01, 95% CI: −22.68 to 10.66; *p* = 0.48). Additionally, a subgroup analysis indicated no significant difference between EA and PUI (WMD: −1.04, 95% CI: −23.59 to 21.52; *p* = 0.93), ED and PUI (WMD: −9.06, 95% CI: −35.20 to 17.08; *p* = 0.50), while a better outcome trend was found in the PUI than SAI group. There was significant between-study heterogeneity (χ^2^ = 11.32, df 4, *p* = 0.02, I^2^ = 65%). No publication bias was detected by Begg’s test (*p* = 0.73).

#### 3.3.4. Mechanical-Activated Irrigation System

**Figure 7:**
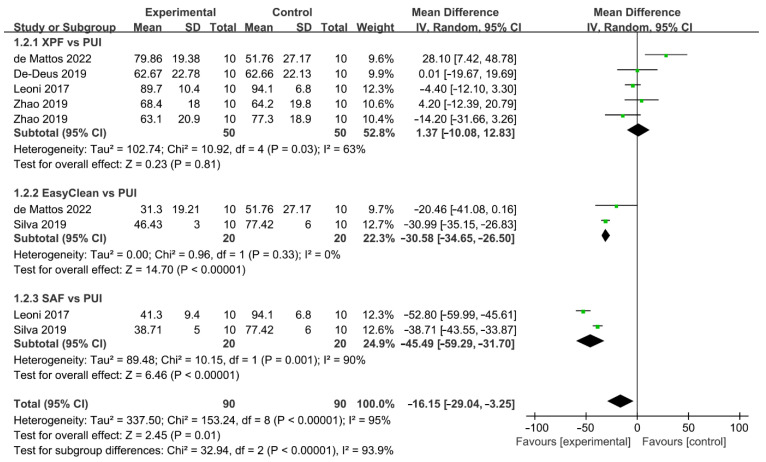
Forest plot and meta-analysis of comparison between mechanical-activated system and PUI [37,38,40,42,43].

A total of five studies described the comparison of the mechanical-activated system and PUI (Figure 7) [37,38,40,42,43]. Five articles with 180 teeth investigated the difference in the effect of AHTD removal between mechanical-activated system and PUI. Zhao included two independent comparisons and each of the other studies included one comparison. Mechanical-activated irrigation system was found to be less effective than PUI and there was statistically significant difference between these two groups (WMD: −16.15, 95% CI: −29.04 to −3.25; *p* = 0.01). A subgroup analysis based on mechanical instruments was carried out. The cleaning efficacy of PUI significantly surpassed EasyClean (WMD: −30.58, 95% CI: −34.65 to −26.50; *p* < 0.00001) and SAF (WMD: −45.49, 95% CI: −59.29 to −31.70; *p* < 0.00001), while the difference between XPF and PUI was not significant (WMD: 1.37, 95% CI: −10.08 to 12.83; *p* = 0.81). There was a significant between-study heterogeneity the in subgroup of XPF vs. PUI (χ^2^ = 10.92, df 4, *p* = 0.03, I^2^ = 63%) and subgroup of SAF vs. PUI (χ^2^ = 10.15, df 1, *p* = 0.001, I^2^ = 90%), and no significant heterogeneity between studies was found in the subgroup of EC vs. PUI (χ^2^ = 0.96, df 1, *p* = 0.33, I^2^ = 0%). No publication bias was detected by Begg’s test (*p* = 0.94).

#### 3.3.5. Laser-Activated Irrigation System

**Figure 8:**
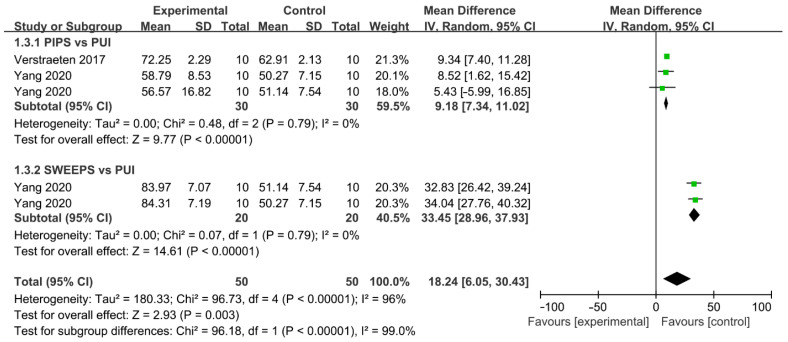
Forest plot and meta-analysis of comparison between LAI and PUI [15,41].

Comparation of percentage reduction in AHTD between LAI and PUI was reported by two studies, with a total of 100 teeth involved (Figure 8) [15,41]. One comparison was included in Verstraeten and two independent comparisons were conducted in Yang. The irrigation efficacy significantly increased in LAI compared with that in PUI (WMD: 18.24, 95% CI: 6.05 to 30.43; *p* = 0.003). A subgroup analysis based on laser-activated instruments was carried out. The cleaning efficacy of PIPS (WMD: 9.18, 95% CI: 7.34 to 11.02; *p* < 0.00001) and SWEEPS (WMD: 33.45, 95% CI: 28.96 to 37.93; *p* < 0.00001) significantly surpassed PUI and SWEEPS, demonstrating the superior efficacy of AHTD removal compared to PIPS. No significant heterogeneity between studies was found in subgroups (χ^2^ = 0.48, df 2, *p* = 0.79, I^2^ = 0%; χ^2^ = 0.07, df 1, *p* = 0.79, I^2^ = 0%) and no publication bias was detected by Begg’s test (*p* = 0.24).

### 3.4. Grading of the Evidence

The confidence rating ranged from very low to low (Supplementary Table S2). All included studies in this meta-analysis were observational studies, which represent a low level of evidence. The confidence ratings in evidence of mechanical vs. PUI, LAI vs. PUI and EV vs. PUI were downgraded because of an indirect outcome of interest.

## 4. Discussion

Due to the limitations of the manual irrigation method, adjunctive root canal irrigation with PUI has been widely applied in the clinic. A review of 28 publications [44] concluded that the combination of SNI and PUI improved the elimination of bacteria and the smear layer throughout the root canal system. However, there are inadequate data about the cleaning efficiency of other irrigation protocols.

### 4.1. Summary of the Main Results

According to this meta-analysis, compared with the control group PUI, LAI was more effective in removing AHTD in root canal treatment; sonically activated irrigation system had similar efficacy, while SNI, negative pressure irrigation system and the mechanical-activated system, especially EasyClean and SAF, were less effective. SWEEPS demonstrated superior efficacy in AHTD removal compared to PIPS. There are multiple potential reasons for the heterogeneity within subgroups, including variations in the operators’ skills and experience, types of teeth, instrumentation techniques and detailed irrigation protocol.

### 4.2. Overall Completeness and Applicability of Evidence

In this study, a comprehensive literature search was conducted using multiple databases. In addition to the five key databases, Google Scholar and the reference lists of included publications were searched manually. Opengrey, OpenDoar, OpenAire and Base were searched for grey studies. 

In this systematic review, the included studies conducted experiments on teeth extracted from people of different ages, genders, ethnicities and races, which means the results of the review might be generalized to patients of diverse demographics. The studies included in our study all employed clinically commonly used NaOCl and EDTA as irrigants, which allows the result to be easily applied in clinical practice. All the research objects in eligible studies included single-canal teeth with low curvature and double-canal teeth with isthmus connection, except one study using C-shaped canals, which means the results of the review might be generalized to different types of root canal morphology. Nevertheless, considering all samples were extracted teeth without pathologic change, teeth with internal absorption, incomplete root formation and root crack should be treated with caution. It is also worth mentioning that all included studies were in vitro experiments and generalizations to practical effects applied in vivo need to be treated with caution.

### 4.3. Agreements and Disagreements with Other Studies or Reviews

#### 4.3.1. Irrigation Protocols

- Passive ultrasonic irrigation (PUI)

To date, five studies compared the AHTD removal efficacy of SNI and PUI using micro-CT. All studies reached consistent conclusions that PUI is more effective. The overall debris reduction after instrumentation and irrigation was observed as 66.81 ± 22.85% by Linden et al. [36], 66.8 ± 29.1% by Rödig et al. [39], 70.75 ± 20% by Zhao et al. [37] and 78.29 ± 33.07% by Rodrigues et al. [35]. Leoni et al. [42] reported a higher debris reduction in 94.1 ± 6.8% after PUI. This might be explained by differences in study design, such as the selection of experimental objects, irrigation solutions, concentration and flow rate of irrigants and the choice of ultrasonic tip. PUI could observably improve the effect of NaOCl [45] because of the stirring effect of ultrasound and the increase in temperature. However, when normal saline was used as the irrigation solution, there was no clear difference in the debris removal effects between PUI and SNI [46]. Some studies evaluated the postoperative pain values in nonvital molars via a visual analog scale (VAS) and they found lower pain values after PUI compared to SNI at all time intervals, especially on the first day, which may indicate a lower amount of extruded irrigant of PUI compared to SNI [47,48]. Our analysis reached a similar result and confirmed the significant advantages of PUI in AHTD removal compared to SNI.

- EndoVac

EndoVac was found to be less effective than PUI regarding to AHTD removal in this meta-analysis and this was inconsistent with the results obtained by the histological method [49,50]. This may be related to the fact that histological studies focused primarily on debris removal in the apical region. Previous studies [12,17,51] reported that the AHTD removal efficacy of EndoVac was better than SNI and PUI in the apical region; specifically, it was better than SNI at 1 mm from WL and comparable to PUI at 1 mm and 3 mm [52]. Our study may imply that the negative pressure system improves the cleaning efficiency of the apical region at the expense of the cleaning efficiency of the pulp cavity and isthmus.

One significant advantage of EndoVac is safety. EndoVac was designed to reduce the risk of extrusion into the periradicular tissues [53]. Endodontic emergencies that occur during or after root canal treatment are often the postoperative pain due to inflammation around the periapical tissues, which is likely to be a result of bacteria, irrigant or filling material being extruded through the apical foramen. Present in vitro studies usually seal the root canal system with 1.5% agar gel, which has similar density with the periodontal tissue (agar: 1045 kg m^−3^ vs. human tissue: 1000–1100 kg m^−3^) [54] to simulate the internal environment. EndoVac has been proved to produce the least debris exclusion in in vitro experiments with the root sealed with the gel [55,56,57]. However, whether the necrotic pulp or presence of periapical lesion has any effect on the apical extrusion of debris is still unclear [53].

- Sonically Activated Irrigation (SAI)

Only three papers used micro-CT to quantify the volume of debris accumulated in the root canal after irrigation using SAI. No statistical difference was observed in this analysis. This is consistent with the evaluation of Rödig et al. [39] and Rodrigues et al. [35]. Linden et al. [36] obtained a superior effect of PUI compared to SAI in removing AHTD. This result might be related to the selection of sonically activated instruments and different ultrasonic power. In these studies, teeth with a curved root canal were selected. The pliable and flexible sonic tip was suitable to extend into the root canal without damaging the integrity of root canal walls. The AHTD removal effect of SAI is commonly similar to SNI in the pulp chamber, but more effective at the apical region, which might be explained by the higher vibration amplitude of the working tip in the apical region [58]. Lower risk of periapical tissue destruction of SAI was reported [59] compared with SNI and PUI.

- Self-adjusting File (SAF)

Two studies compared the reduction in AHTD by micro-CT after application of SAF and PUI. SAF was reported to be more efficient in removing AHTD and bacteria compared with SNI [60,61]. The AHTD removal efficacy was not as good as PUI [62]. Despite the Ni-Ti instrument being hollow and flexible, it inevitably generates the continuous gentle pressure on the canal walls and produces new debris during the irrigation process. Further, it is hard for SAF to enter complex structures, such as the isthmus area [63].

- XP-endo Finisher (XPF)

Three studies compared the reduction in AHTD by micro-CT after application of XPF and PUI. No difference was found in any of these studies and this was consistent with another result obtained by SEM [27]. The effect of biofilm removal of XPF and PUI showed no difference as well [64,65]. The manufacturer of XP-endo Finisher recommended its use for the chemo-mechanical preparation of highly complex root canals, such as internal root resorption cavities. Ulusoy et al. [66] proved that XPF was more effective than PUI to remove organic tissue from artificial internal resorption cavities. However, the removal of calcium hydroxide in artificial internal resorption cavities showed no significant difference [67].

- PIPS

Two studies compared the reduction in AHTD after application of PIPS and PUI by the use of micro-CT. Both studies led to the result that PIPS was superior to PUI. The result was consistent with the outcomes performed by digital images obtained using a stereomicroscope [68,69]. This might be explained by three aspects of PIPS: (i) the induced peak velocity is significantly higher; (ii) the irrigant can be delivered deeper into the lateral canal; and (iii) the induced flow field in PIPS implies a periodic change in the sign of the wall-shear stress during the back-and-forth flush [70]. Despite this technique only requiring placing the laser tip in the pulp chamber, the safety of PIPS was still the main drawback. Some studies pointed out that PIPS produced the most debris exclusion in in vitro experiments [71].

#### 4.3.2. Complexity of Root Canal Anatomy

The cleaning efficacy of the same irrigation method can vary greatly among different studies. This may be related to the anatomical complexity of root canals. For example, incisors often present flat roots and canal systems, especially in the apical third region [20]. Therefore, the root canal walls may not be completely covered by the instrument in a specific region [72,73]. The same principle is applied to other complex root canal anatomy, such as isthmus and C-shaped root canals. For irrigation protocols that mostly rely on mechanical properties, such as XPF, the soft Ni-Ti file may not fully adapt to the shape of the complex root canal anatomy, while irrigation protocols that mostly rely on the fluid dynamics are not affected as much. 

The AHTD removal efficacy is also inseparable from the curvature of root canals. For example, the percentage reduction in AHTD after using XPF was measured 53.65% in 25–35° curved root canals [29] and 69.57% in 10–20° curved canals [38]. It has been proved that the canal curvature negatively affects the cleaning efficacy of irrigation methods [74]. In the same article, the effect was found to be most pronounced for the sonic techniques, while not the case for PUI. This could be ascribed to the fact that the pre-bending ultrasonic tip was adapted to the shape of root canals while the vibration of the sonic file was restricted by severe curved canals.

#### 4.3.3. Application of Root Canal Irrigants

NaOCl is the most widely used irrigant in the clinic because of its broad antibacterial spectrum and capacity to dissolve the bacteria film and residual pulp tissue. The antibacterial ability obviously declined with a decrease in solution concentration [75]. Pulp tissue can dissolve quickly and thoroughly when it is inserted into a test tube containing NaOCl in vitro. In that case, the volume of NaOCl is infinitely larger than that of dental pulp and the inactivation of NaOCl is negligible [76,77]. When applied in the root canal, NaOCl has a finite volume and is consumed and inactivated quickly. Therefore, the irrigation solution needs to be renewed frequently to ensure its activity [78]. Massive dosage and sufficient application time of the irrigant are considered to promote the intracanal disinfection and debridement [79]. However, another study showed that the impact of dosage and application time of NaOCl is negligible, because extensive irrigation and frequent exchange can compensate for solution inactivation [80]. There are controversial debates about the appropriate concentration and application time of NaOCl for root canal irrigation. The dosage and the application time cannot be raised arbitrarily. Higher dose and longer application time were found to negatively influence the fracture resistance of endodontically treated teeth [81,82,83].

Ethylene diamine tetraacetic acid (EDTA) is a calcium-chelating agent. The 17% neutralized solution is commonly used to remove inorganic components from the smear layer and help reduce AHTD from root canals. Final irrigation followed by EDTA was proved to remove more hard tissue debris than that followed by normal saline [3,84]. The concentration of EDTA has no significant effect on the removal of smear layer and debris [85]. EDTA was used after final irrigation in five articles in our research and no obvious difference was found compared to the studies that EDTA was not used.

In the clinic, multiple irrigants are often used in combination. The order of irrigants is considered to influence the cleaning effect. The available chlorine is crucial for NaOCl to dissolve bacteria and infected tissue. The chemical interaction between EDTA and NaOCl decreases the supply of available chlorine. While the presence of NaOCl had little effect on the calcium-chelating ability of EDTA [86], NaOCl increases the removal of the organic phase from mineralized dentin and promotes the exposure of inorganic substrates, which help EDTA penetrate the intratubular and peritubular dentine to expedite their disintegration [82,87]. Therefore, sequences of NaOCl/EDTA or NaOCl/EDTA/NaOCl are usually recommended.

### 4.4. Strengths and Limitations

The present analysis summarized the current evidence on the relationship between activated irrigation protocols and AHTD removal. Several main databases were searched thoroughly by two independent reviewers and detailed inclusion criteria were adopted to ensure the accuracy and integrity of included literature. The volume of AHTD in the included studies was quantified by micro-CT to minimize subjective judgment bias and brought high-confidence evidence. Furthermore, the random-effects model was applied to reduce the heterogeneity among some subgroups, which may lead to doubts about the results.

The present meta-analysis does have some limitations. First, the main limitation is that all the included studies were observational studies, with a low level of evidence, which may affect the quality of the results. Among these, only one article mentioned the removal of AHTD in different areas (coronal third, middle third, apical third), which may be of great importance in root canal therapy. Additionally, language was limited to English during the literature screening process, which may increase publication bias. Finally, this meta-analysis was conducted at the literature level; thus, individual factors cannot be avoided.

## 5. Prospective

The present study indicates that PUI does have prominent advantages in removing AHTD compared with SNI. SWEEPS and PIPS demonstrate better performance than PUI in our analysis. However, high-quality experimental data are still limited at present and further well-designed investigations are warranted to provide evidence-based recommendation for clinical practice. 

For future studies in this topic, firstly, more well-designed studies can be conducted to further explore the effect of LAI and its comparison with PUI. Secondly, a standardized irrigation procedure with PUI as the control group can be developed and adopted to minimize the bias and heterogeneity. Additionally, some studies assessed irrigation efficacy on filling remnant removal using micro-CT [88,89,90,91,92,93,94]. The meta-analysis of the percentage reduction in calcium hydroxide or gutta-percha after irrigation assessed by micro-CT can be performed for comprehensive evaluation of various irrigation techniques applied in different scenarios.

## 6. Conclusions

This meta-analysis indicates that PUI is superior to SNI for debris removal; SWEEPS and PIPS has better AHTD removal performance than PUI. This result suggests that SWEEPS and PIPS may be a better option for hard-to-clean root canals. In contrast, the negative pressure system and mechanical-activated system were shown to be less effective, which may need to be selected under appropriate indications and require longer activation times to achieve satisfactory clinical outcomes.

## Figures and Tables

**Table 1:** Main characteristics of included studies.

Study ID	Subjects	Total Sample	Root Canal Irrigation			Outcome of Interest
			Groups	Irrigation Protocols	Irrigation Solutions	
Freire 2015	Mesial canals of mandibular molars, with a curvature of 25–35°	24	PUI (*n* = 12)	At power 5 (Suprasson P5, France), A #20 Irrisafe ultrasonic tip, with an in-and-out motion, 2 mm away from the WL	2 mL 1% NaOCl (30 s) + 2 mL 17%EDTA (30 s) + 2 mL 1% NaOCl (30 s)	Reduction in debris (%) 55.55 ± 21.91
			EV (*n* = 12)	Each cycle repeating the movement of microcannula: 1 mm from the WL for 6 s, followed by withdrawal to 2 mm from the WL for 6 s	2 mL 1% NaOCl (30 s) + 2 mL 17%EDTA (30 s) + 2 mL 1% NaOCl (30 s)	Reduction in debris (%) 53.65 ± 18.16
Leoni 2017	Mesial canals of mandibular first molars, with a curvature of 15–20° Two mesial canals connected by a single and continuous isthmus that joined together in the apical third to exit in a single foramen	40	PUI (*n* = 10)	35 Hz (10% power of Piezon 150), A #20 Irrisonic ultrasonic tip, with an in-and-out motion, 2 mm away from the WL	0.5 mL 2.5% NaOCl (20 s) + 1.67 mL 2.5% NaOCl (20 s) + 1.67 mL 2.5% NaOCl (20 s) + 1.67 mL 2.5% NaOCl (20 s)	Reduction in debris (%) 94.1 ± 6.8
			XPF (*n* = 10)	Instrument inserts without rotation, then turns on rotation. With an in-and-out motion, (800 rpm, 1 N·cm) Up to the WL.	0.5 mL 2.5% NaOCl (60 s) + 5 mL 2.5% NaOCl (SNI, 60 s)	Reduction in debris (%) 89.7 ± 10.4
			SNI (*n* = 10)	30-gauge NaviTip needle, 2 mm away from the WL	0.5 mL 2.5% NaOCl (left still for 60 s) + 5 mL 2.5% NaOCl (60 s)	Reduction in debris (%) 45.7 ± 15
			SAF (*n* = 10)	A 1.5-mm diameter SAF file (ReDent-Nova). With an in-and-out motion, Up to the WL	0.5 mL 2.5% NaOCl (left still for 60 s) + 5 mL 2.5% NaOCl (60 s)	Reduction in debris (%) 41.3 ± 9.4
Verstraeten 2017	Mesial canals of mandibular molars. Two mesial canals connected by an isthmus	30	PUI (*n* = 10)	At power 4 (Suprasson Pmax), A #20 Irrisafe ultrasonic tip, 2–4 mm away from the WL	1 mL 2.5% NaOCl (20 s) + 1 mL 2.5% NaOCl (20 s) + 1 mL 2.5% NaOCl (20 s)	Vol% debris after preparation (%) 8.44 ± 2.15 Vol% debris after irrigation (%) 3.13 ± 0.98
			LAI (*n* = 10)	A 2940 nm Er:YAG laser (AT Fidelis) (Energy:20 mJ; frequency:20 Hz; length:50 μs) A 300 μm diameter tip (PRECISO 300/14). With an in-and-out motion. 5 mm away from the WL.	1 mL 2.5% NaOCl (20 s) + 1 mL 2.5% NaOCl (20 s) + 1 mL 2.5% NaOCl (20 s)	Vol% debris after preparation (%) 8.21 ± 1.77 Vol% debris after irrigation (%) 2.43 ± 0.91
			PIPS (*n* = 10)	A 2940 nm Er:YAG laser (AT Fidelis) (Energy:20 mJ; frequency:20 Hz; length:50 μs). A 300-μm tip (PRECISO 300/14). Held still at the canal entrance. 5 mm away from the WL.	1 mL 2.5% NaOCl (20 s) + 1 mL 2.5% NaOCl (20 s) + 1 mL 2.5% NaOCl (20 s)	Vol% debris after preparation (%) 8.18 ± 2.11 Vol% debris after irrigation (%) 2.28 ± 1.05
De-Deus 2019	Mandibular incisors with a single oval-shaped canal	20	XPF (*n* = 10)	Instrument inserts without rotation, then turns on rotation. With an in-and-out motion. (800 rpm, 1 N·cm) Up to the WL.	0.5 mL 5.25% NaOCl (60 s) + 4.5 mL 5.25% NaOCl (60 s, SNI at 1 mm from the WL)	Reduction in debris (%) 62.67 ± 22.78
			PUI (*n* = 10)	35 Hz (10% power of Piezon 150), A #20 Irrisonic ultrasonic tip, With an in-and-out motion, 2 mm away from the WL	0.5 mL 5.25% NaOCl (20 s) + 1.5 mL 5.25% NaOCl (20 s) + 1.5 mL 5.25% NaOCl (20 s) + 1.5 mL 5.25% NaOCl (20 s)	Reduction in debris (%) 62.66 ± 22.13
Rödig 2019	Mesial canals of mandibular molars, with a curvature of 15–20° and a radius between 5.5 and 16.5 mm. Two mesial canals connected by an isthmus	40	EA (*n* = 10)	166 Hz, A # 15 EndoActivator tip, 2 mm away from the WL	1 mL 1% NaOCl (20 s) + 2 mL 1% NaOCl (20 s) + 2 mL 1% NaOCl (20 s) + 2 mL 15% EDTA (20 s)	Reduction in debris (%) 54.1 ± 21.5
			ED (*n* = 10)	6000 Hz, An EDDY tip (VDW), 2 mm away from the WL	1 mL 1% NaOCl (20 s) + 2 mL 1% NaOCl (20 s) + 2 mL 1% NaOCl (20 s) + 2 mL 15% EDTA (20 s)	Reduction in debris (%) 56.9 ± 24.2
			PUI (*n* = 10)	30% power of VDW Ultra, A #25 IRRI S ultrasonic tip, 2 mm away from the WL	1 mL 1% NaOCl (20 s) + 2 mL 1% NaOCl (20 s) + 2 mL 1% NaOCl (20 s) + 2 mL 15% EDTA (20 s)	Reduction in debris (%) 66.80 ± 29.10
			SNI (*n* = 10)	A 30-gauge Endo-EZE needle (Ultradent), 2 mm away from the WL	1 mL 1% NaOCl (20 s) + 2 mL 1% NaOCl (20 s) + 2 mL 1% NaOCl (20 s) + 2 mL 15% EDTA (20 s)	Reduction in debris (%) 44.10 ± 17.40
Silva 2019	Mesial canals of mandibular molars, with a curvature of 10–20° and isthmuses type I or III	40	PUI (*n* = 10)	10% power of Piezo, A #20/0.01 Irrisonic ultrasonic tip, 1 mm away from the WL	4 mL 5.25% NaOCl (30 s) + 4 mL 5.25% NaOCl (30 s) + 4 mL 5.25% NaOCl (30 s) + 4 mL 17%EDTA (30 s) + 4 mL 5.25% NaOCl (30 s)	Vol% debris after preparation (%) 0.63 ± 1.56 Vol% debris after irrigation (%) 0.14 ± 0.38
			EV (*n* = 10)	First: EndoVac microcannula was inserted into the root canal until finding resistance and moved up and down. Then: EndoVac microcannula was inserted 1 mm short of the WL.	6 mL 5.25% NaOCl (activation for 30 s with microcannula, then left still for 1 min) + 5 mL 5.25% NaOCl (activation for 60 s with microcannula, then left still for 1 min) + 4 mL 17%EDTA (ditto) + 5 mL 5.25% NaOCl (ditto)	Vol% debris after preparation (%) 0.46 ± 0.73 Vol% debris after irrigation (%) 0.14 ± 0.27
			SAF (*n* = 10)	A 2 mm-diameter SAF file (ReDent-Nova). With an in-and-out motion. 1 mm away from the WL.	12 mL 5.25% NaOCl (3 min) + 4 mL 17%EDTA (1 min) + 4 mL 5.25% NaOCl (1 min)	Vol% debris after preparation (%) 0.32 ± 0.55 Vol% debris after irrigation (%) 0.20 ± 0.40
			Easy-Clean (*n* = 10)	1 mm away from the WL.	Similar with PUI	Vol% debris after preparation (%) 0.30 ± 0.34 Vol% debris after irrigation (%) 0.17 ± 0.28
Zhao 2019	Mandibular molars with a C-shaped canal system	60	SNI (*n* = 20)	A 30-gauge needle. 1 mm away from the WL.	2 mL 2% NaOCl × 3 (SNI at a rate of 5 mL/min, then left still for 20 s) + 2 mL 17% EDTA (5 mL/min) + 2 mL 2% NaOCl (5 mL/min)	Reduction in debris (%) Group1: 43.4 ± 17.8 Group2: 57.1 ± 20.6
			PUI (*n* = 20)	At power 6 (Suprasson P5, France). A #20 Irrisafe ultrasonic tip With an in-and-out motion. 1 mm away from the WL.	2 mL 2% NaOCl (20 s) × 3 + 2 mL 17% EDTA (SNI, 5 mL/min) + 2 mL 2% NaOCl (SNI, 5 mL/min)	Reduction in debris (%) Group1: 64.2 ± 19.8 Group2: 77.3 ± 18.9
			XPF (*n* = 20)	A #25/.00 XPF file inserts without rotation, then turns on rotation. With an in-and-out motion. (800 rpm, 1 N·cm) Up to the WL.	1 mL 2% NaOCl (SNI at 1 mm away from the WL, 5 mL/min) + 5 mL 2% NaOCl (XPF for 1 min) + 2 mL 17% EDTA (SNI, 5 mL/min) + 2 mL 2% NaOCl (SNI, 5 mL/min)	Reduction in debris (%) Group1: 68.4 ± 18.0 Group2: 63.1 ± 20.9
Linden 2020	Mesial canals of mandibular molars with a moderate curvature. Two mesial canals connected by an isthmus.	27	SNI (*n* = 9)	A 30-G notched needle. 2 mm away from the WL.	3 mL 2.5% NaOCl (0.14 mL/s)	Reduction in debris (%) 43.68 ± 30.09
			ED (*n* = 9)	6000 Hz. A #25/.04 EDDY tip. 2 mm away from the WL.	1 mL 2.5% NaOCl (20 s) × 3	Reduction in debris (%) 36.38 ± 20.43
			PUI (*n* = 9)	At power 9 (Suprasson P5, France). A #20 Irrisafe ultrasonic tip. Without an in-and-out motion. 2 mm away from the WL.	1 mL 2.5% NaOCl (20 s) × 3	Reduction in debris (%) 66.81 ± 22.85
Yang 2020	Mandibular molars, with a single canal in the distal root and two mesial canals connected by an isthmus and have a curvature of 25–35°	30	PUI (*n* = 10)	At power 5 (Suprasson P5, France). A #15/.02 Irrisafe ultrasonic tip. With an in-and-out motion. 2 mm away from the WL.	0.5 mL 1% NaOCl (SNI) + 5 mL 1% NaOCl (activation for 30 s, then left still for 30 s) × 3	Reduction in debris (%) Group1: 50.27 ± 7.15 Group2: 51.14 ± 7.54
			PIPS (*n* = 10)	A 2940 nm Er:YAG laser (LightWalker AT) (Energy:20 mJ; frequency:15 Hz; length:50μs). A 600 μm diameter tip (PIPS 600/9). Held still at the canal entrance	0.5 mL 1% NaOCl (SNI) + 5 mL 1% NaOCl (activation for 30 s, then left still for 30 s) × 3	Reduction in debris (%) Group1: 58.79 ± 8.53 Group2: 56.57 ± 16.82
			SWEEPS (*n* = 10)	A 2940 nm Er:YAG laser (LightWalker AT) (Energy:20 mJ; frequency:15 Hz; length:50 μs). A special fibre tip (SWEEPS 600).	0.5 mL 1% NaOCl (SNI) + 5 mL 1% NaOCl (activation for 30 s, then left still for 30 s) × 3	Reduction in debris (%) Group1: 84.31 ± 7.19 Group2: 83.97 ± 7.07
Rodrigues 2021	Mesial canals of mandibular molars, with a curvature of 20–46° (mean 32.5°) and isthmuses type I	24	PUI (*n* = 8)	At medium power of ultrasonic unit (SEM). An ultrasonic ESI Tip (SEM). 2 mm away from the WL.	5 mL 3% NaOCl (20 s) + 5 mL 17% EDTA (20 s) + 5 mL 3% NaOCl (20 s) + 5 mL saline solution (SNI)	Reduction in debris (%) 78.29 ± 33.07
			SNI (*n* = 8)	A 30-G Navitip needle 1 mm away from the WL.	5 mL 3% NaOCl (20 s) + 5 mL 17% EDTA (20 s) + 5 mL 3% NaOCl (20 s) + 5 mL saline solution	Reduction in debris (%) 56.98 ± 46.45
			ED (*n* = 8)	An EDDY tip. 1 mm away from the WL.	5 mL 3% NaOCl (20 s) + 5 mL 17% EDTA (20 s) + 5 mL 3% NaOCl (20 s) + 5 mL saline solution (SNI)	Reduction in debris (%) 93 ± 6.14
de Mattos 2022	mesial roots of mandibular molars, with a curvature of 10–20° and isthmuses type II	40	PUI (*n* = 10)	15% power of Jet Sonic (Brazil). A #20 Irrisafe ultrasonic tip. 1 mm away from the WL.	2 mL 2.5% NaOCl (1 min) + 2 mL 17% EDTA (1 min)	Reduction in debris (%): 51.76 ± 27.17
			XPF (*n* = 10)	An XPF file inserts without rotation, then turns on rotation. (800 rpm, 1 N·cm) 1 mm away from the WL.	0.5 mL 5.25% NaOCl (1 min) +4.5 mL 5.25% NaOCl (SNI)	Reduction in debris (%): 79.86 ± 19.38
			EA (*n* = 10)	A #25/04 EndoActivator tip, 1 mm away from the WL, activated at 10,000 cycles per minute.	2 mL 2.5% NaOCl (1 min) + 2 mL 17% EDTA (1 min) 5 mL 2.5% NaOCl (1 min) × 2 + 5 mL 17% EDTA (1 min) + 5 mL 2.5% NaOCl (1 min) 5 mL 2.5% NaOCl (microcannula) + 5 mL 2.5% NaOCl (microcannula) × 3	Reduction in debris (%): 62.08 ± 22.36
			EasyClean (*n* = 10)	1 mm away from the WL		Reduction in debris (%): 31.30 ± 19.21

PUI: Passive ultrasonic irrigation. EV: EndoVac. XPF: XP-Endo Finisher. SNI: Syringes and needles irrigation. SAF: Self-adjusting File. LAI: Laser Activated Irrigation. PIPS: Photon Induced Photoacoustic Streaming. EA: Endoactivator. ED: EDDY. SWEEPS: Shock-Wave Enhanced Emission Photoacoustic Streaming.

## Data Availability

The data presented in this study are available in Supplementary Materials.

## Supplementary Materials

The following supporting information can be downloaded at: https://www.mdpi.com/article/10.3390/jcm11206053/s1, Table S1: Detailed searching strategy; Table S2: Grading of the evidence; Excel S1: Data collection.
