# Supplementary material for: Micro-CT Evaluation of Different Root Canal Irrigation Protocols on the Removal of Accumulated Hard Tissue Debris: A Systematic Review and Meta-Analysis
Source: J Clin Med. 2022 Oct 13;11(20):6053. doi: 10.3390/jcm11206053 (PMC9605157; doi:10.3390/jcm11206053)
Supplement: Supplementary file 1 [file jcm-11-06053-s001.zip › Supplementary Materials.pdf]

Supplementary Table S1. Detailed searching strategy.

Database	Search String
Pubmed	(((((root canal therapy[MeSH Terms]) OR (canal)) OR (endodontic)) AND ((((((((micro ct, x ray[MeSH Terms])) OR (X Ray Microtomography)) OR (MicroCT)) OR (MicroCTs)) OR (Microcomputed Tomography)) OR (Tomography, Microcomputed)) OR (computed tomography))) AND (((((((therapeutic irrigation[MeSH Terms]) OR (irrigation)) OR (irrigations)) OR (Lavage)) OR (Lavages)) OR (Douching)) OR (Douchings))) AND (((((hard tissue debris) OR (ahtd)) OR (htd)) OR (debris)) OR (debridement))
Web of Science	(TS=(Canal) OR TS=(endodontic)) AND (TS=(X Ray Microtomography) OR TS=(Microtomography, X-Ray) OR TS=(MicroCT) OR TS=(MicroCTs) OR TS=(Microcomputed Tomography) OR TS=(Tomography, Microcomputed) OR TS=(computed tomography)) AND (TS=(Therapeutic Irrigation) OR TS=(Irrigation, Therapeutic) OR TS=(irrigation) OR TS=(irrigations) OR TS=(Lavage) OR TS=(Lavages) OR TS=(Douching) OR TS=(Douchings)) AND (TS=(hard tissue debris) OR TS=(AHTD) OR TS=(HTD) OR TS=(debris) OR TS=(debridement))
Embase	('endodontic procedure'/exp OR 'canal' OR 'endodontic') AND ('micro-computed tomography'/exp OR 'micro-ct' OR 'microct' OR 'microcts' OR 'micro-cts' OR 'microcomputed tomography' OR 'micro computed tomography') AND ('lavage'/exp OR 'irrigation' OR 'irrigations' OR 'lavage' OR 'lavages' OR 'douching' OR 'douchings') AND ('dental surgery'/exp OR 'hard tissue debris' OR 'ahtd' OR 'htd' OR 'debris' OR 'debridement')
Cochrane	#1 MeSH descriptor: [Root Canal Therapy] this term only<br>#2 Canal or Endodontic<br>#3 #1 or #2<br>#4 MeSH descriptor: [Therapeutic Irrigation] this term only<br>#5 Therapeutic Irrigation or Irrigation, Therapeutic or irrigation or irrigations or Lavage or Lavages or Douching or Douchings<br>#6 #4 or #5<br>#7 MeSH descriptor: [X-Ray Microtomography] this term only<br>#8 micro-ct or micro-cts or microct or microcts or microcomputed tomography or micro computed tomography or micro-computed tomography<br>#9 #7 or #8<br>#10 MeSH descriptor: [Smear Layer] this term only<br>#11 hard tissue debris or ahtd or htd or debris or debridement or gutta percha or guttapercha or gutta-percha or ultrafil or thermafil<br>#12 #10 or #11<br>#13 #3 and #6 and #9 and #12
Scopus	(ALL (Canal OR endodontic) AND ALL (irrigation OR irrigations OR lavage OR lavages OR douching OR douchings) AND ALL (micro-ct OR micro-cts OR microct OR microcts OR "microcomputed tomography" OR "micro computed tomography" OR "micro-computed tomography") AND ALL ("hard tissue debris" OR ahtd OR htd OR debris OR debridement))

Supplementary Table S2. Grading of the evidence.

Certainty assessment							№ of patients		Effect		Certainty	Importance
№ of studies	Study design	Risk of bias	Inconsistency	Indirectness	Imprecision	Other considerations	New Comparison	placebo	Relative (95% CI)	Absolute (95% CI)		
**SAI vs PUI**												
4	observational studies	not serious	not serious	not serious	not serious	none	47	47	-	MD **6.01 lower** (22.68 lower to 10.66 higher)	⊕⊕◯◯ Low	IMPORTANT
**SAI vs PUI - EA vs PUI**												
2	observational studies	not serious	not serious	not serious	not serious	none	20	20	-	MD **1.04 lower** (23.59 lower to 21.52 higher)	⊕⊕◯◯ Low	IMPORTANT
**SAI vs PUI - EDDY vs PUI**												
3	observational studies	not serious	not serious	not serious	not serious	none	27	27	-	MD **9.06 lower** (35.2 lower to 17.08 higher)	⊕⊕◯◯ Low	IMPORTANT
**Mechanical vs PUI**												
5	observational studies	not serious	not serious	not serious	serious[a]	none	90	90	-	MD **13.3 lower** (33.84 lower to 7.24 higher)	⊕◯◯◯ Very low	IMPORTANT
**Mechanical vs PUI - XPF vs PUI**												
4	observational studies	not serious	not serious	not serious	not serious	none	50	50	-	MD **1.37 higher** (10.08 lower to 12.83 higher)	⊕⊕◯◯ Low	IMPORTANT
**Mechanical vs PUI - EasyClean vs PUI**												
2	observational studies	not serious	not serious	not serious	serious[a]	none	20	20	-	MD **23.46 lower** (41.74 lower to 5.17 lower)	⊕◯◯◯ Very low	IMPORTANT
**Mechanical vs PUI - SAF vs PUI**												
2	observational studies	not serious	not serious	not serious	serious[a]	none	20	20	-	MD **52.61 lower** (59.75 lower to 45.48 lower)	⊕◯◯◯ Very low	IMPORTANT
**LAI vs PUI**												
2	observational studies	not serious	not serious	not serious	serious[a]	none	50	50	-	MD **18.38 higher** (5.76 higher to 31 higher)	⊕◯◯◯ Very low	IMPORTANT
**LAI vs PUI - PIPS vs PUI**												
2	observational studies	not serious	not serious	not serious	serious[a]	none	30	30	-	MD **8.26 higher** (3.48 higher to 13.04 higher)	⊕◯◯◯ Very low	IMPORTANT
**LAI vs PUI - SWEEPS vs PUI**												
1	observational studies	not serious	not serious	not serious	not serious	none	20	20	-	MD **33.45 higher** (28.96 higher to 37.93 higher)	⊕⊕◯◯ Low	IMPORTANT
**EV vs PUI**												
2	observational studies	not serious	not serious	not serious	serious	none	22	22	-	MD **3.63 lower** (17.35 lower to 10.08 higher)	⊕◯◯◯ Very low	IMPORTANT
**SNI vs PUI**												
5	observational studies	not serious	not serious	not serious	not serious	none	57	57	-	MD **27.89 higher** (14.61 higher to 41.17 higher)	⊕⊕◯◯ Low	IMPORTANT

(CI: confidence interval; MD: mean difference; a. One study only report the volume of debris obtained before (A) and after (B) the final irrigation, the percentage debris reduction (red%) was calculated according to the following formula: red% = 100 * (A − B) / A.
